# Edge-aware GAT-based protein binding sites prediction

**DOI:** 10.1371/journal.pcbi.1014552

**Published:** 2026-08-11

**Authors:** Weisen Yang, Hanqing Zhang, Wangren Qiu, Xuan Xiao, Weizhong Lin

**Affiliations:** 1 School of Information Engineering, Jingdezhen Ceramic University, Jingdezhen, China; 2 School of Information Engineering, Jiangxi Art and Ceramics Technology Institute, Jingdezhen, China; Xinjiang Technical Institute of Physics and Chemistry, CHINA

## Abstract

Accurate identification of protein binding sites is crucial for understanding biomolecular interaction mechanisms and for the rational design of drug targets. Traditional predictive methods often struggle to balance prediction accuracy with computational efficiency when capturing complex spatial conformations. To address this challenge, we propose an Edge-aware Graph Attention Network (Edge-aware GAT) model for the fine-grained prediction of binding sites across various biomolecules, including proteins, DNA/RNA, ions, ligands, and lipids. Our method constructs atom-level graphs and integrates multidimensional structural features, including geometric descriptors, DSSP-derived secondary structure, and relative solvent accessibility (RSA), to generate spatially aware embedding vectors. By incorporating interatomic distances and unit direction vectors as geometric edge features within the attention mechanism, the model enhances local structural representation without claiming strict SE(3)- or E(3)-equivariance. On benchmark datasets, our model achieves a ROC-AUC of 0.93 for protein-protein binding site prediction and shows competitive performance among the evaluated structure-based atomic-level models. The use of geometry-aware edge attention and residue-level attention pooling further improves both binding site localization and the capture of local structural details. Visualizations using PyMOL confirm the model’s practical utility and interpretability. To facilitate community access and application, we have deployed a publicly accessible web server at http://119.45.201.89:5000/. In summary, our approach offers an efficient structure-based framework that balances prediction accuracy, generalization, and interpretability for identifying functional sites in proteins.

## Author summary

Predicting where and how proteins interact with other molecules is fundamental to understanding life at the molecular level and for developing new drugs. However, traditional computational methods often struggle to capture the complex three-dimensional shapes of proteins, making it difficult to accurately identify these critical binding sites. In this study, we introduce an Edge-aware Graph Attention Network (Edge-aware GAT) that treats proteins as detailed atomic-level graphs. Unlike methods that rely only on scalar distance information or coarse residue-level representations, our model uses both interatomic distances and unit direction vectors as geometric edge features to guide attention-based neighborhood aggregation. This design should be interpreted as geometry-aware edge attention rather than strict rotation-equivariant tensor propagation. We demonstrate that the proposed model achieves competitive performance among evaluated structure-based atomic-level methods for identifying binding sites across several molecular partners, including other proteins, DNA/RNA, ions, ligands, and lipids. To make this tool accessible to the broader research community, we have also deployed a freely available web server where users can upload protein structures and obtain predictions. By improving accuracy and interpretability within this evaluated benchmark setting, our work provides a practical tool for structural biology and rational therapeutic design.

## Introduction

Proteins are fundamental components of life, regulating most biological functions through their specific molecular interactions. In biological systems, molecular interfaces are ubiquitous, playing a central role in the formation of cellular boundaries and intracellular organization. However, their functional significance extends beyond these roles. Proteins exert their biological functions by interacting with other proteins, nucleic acids, cell membranes, various small molecules, and ions. The analysis and comparison of protein binding sites are critical for a wide range of applications in drug discovery, including de novo drug identification, drug repurposing, and polypharmacology [1]. Understanding the structure and properties of proteins—especially their binding sites—is essential for elucidating their biological functions and for developing therapeutic agents. Computational techniques have become indispensable in the drug discovery process, enabling approaches such as virtual screening of small molecules [2], structure-based drug design, and molecular docking between small molecules and proteins. Studies analyzing ligand-protein complexes in the Protein Data Bank (PDB) [3] have shown that most ligands interact with specific binding sites on their target proteins. Each of these binding sites is characterized by a unique set of properties or biological functions that distinguish them from other surface cavities on the protein [4].

Predicting whether a given protein can interact with other molecules remains a significant challenge in biology. Despite substantial progress in various areas, this problem is still far from being fully solved [5]. Many machine learning-based methods have been developed for protein interaction site prediction [6–17], further expanding the methodological options in this field. These earlier studies laid the foundation for many current approaches, exploring various machine learning algorithms and feature extraction techniques. However, the field has advanced considerably since then, with newer methods incorporating deep learning and sophisticated structural analysis to achieve greater accuracy and efficiency.

The emergence of deep learning techniques has led to the development of several effective approaches for protein binding site prediction. These include methods based on spherical convolutional neural networks such as Spherical CNN [18], DeepSphere [19], geometric deep learning-based frameworks like MaSIF, which predicts molecular surface interactions [20], structure-based predictors such as ScanNet [21], and parameter-free geometric learning models like PeSTo, which target protein interaction interfaces [22]. These methods have demonstrated promising results across diverse large-scale datasets. However, despite their importance, such experimental and computational techniques often come with high costs, significant computational complexity, long runtimes, and complicated operational procedures. Specifically, computing protein surface representations and extracting surface features are time-consuming and technically challenging. Furthermore, surface parameterization is usually sensitive to structural details and errors in protein models [23]. Therefore, developing an efficient and robust computational framework for predicting protein binding sites remains an urgent and valuable task.

Early representative models such as DeepSite [24], CNNSite [25], CNN-LSTM [26], and Frsite [27] demonstrated that CNNs and LSTMs could effectively learn complex protein patterns. In recent years, deep learning techniques—particularly those that incorporate structural information—have demonstrated tremendous potential in modeling molecular interactions within the field of bioinformatics. With the emergence of Graph Convolutional Networks (GCNs) in structural analysis of proteins, researchers began to leverage these advanced neural architectures to process three-dimensional protein structural data. Graph Neural Networks (GNNs) [28] are particularly well-suited for modeling the structural and interactional properties of proteins, as amino acid chains often exhibit complex spatial configurations that naturally form graph-like structures. By applying graph convolution, it is possible to effectively capture both local structural features and global topological information, thereby significantly improving the accuracy of binding site prediction. Zitnik et al. demonstrated the use of GNNs for modeling protein interactions and structural representations with promising results [29]. Building on this, Ryu developed an optimized GNN-based model for protein-ligand binding prediction, enhancing both accuracy and computational efficiency [30]. Furthermore, studies by Yuan [31] and Zaki [32] integrated protein spatial structures into GNN frameworks to further improve the prediction of functional sites. Recent graph-representation and graph-clustering studies have further broadened the methodological context of GNN-based biomedical modeling: Su et al. introduced an interpretable transformer-powered graph representation framework for biological networks [33],Yang et al. developed a multi-view neural fusion strategy for protein complex identification [34], and Yang et al. proposed link-based attributed graph clustering to jointly exploit links and node attributes [35]. These studies collectively emphasize the importance of integrating graph topology, attribute information, and model interpretability, which is consistent with the design motivation of our edge-aware atomic graph framework. Compared to traditional Convolutional Neural Networks (CNNs), GCNs are inherently more capable of handling non-Euclidean data structures, making them especially advantageous for modeling complex biomolecules like proteins with intricate spatial relationships.

Many successful approaches for protein binding site prediction have combined Transformer architectures with geometric deep learning [20], representing protein structures as graphs or point clouds. These methods leverage the translation-invariance properties of neural networks to model protein geometry and spatial interactions effectively [36–38]. Building on these principles, two mainstream paradigms have emerged: voxel-based and graph-based representations, each with distinct computational strategies. Voxel-based methods discretize protein structures into 3D grids, enabling direct application of Conventional Neural Networks (CNNs). OctSurf [39] and 3D U-Net [40] pioneered this approach by utilizing CNN to voxel grids for simultaneous detection of surface and interior binding sites. Similarly, Pinheiro [32] proposed a voxel-based CNN for drug design, while VoxPred [41] enhanced the paradigm by combining raw 3D structural data with voxel grids, achieving improved predictive performance across multiple datasets. Collectively, these models demonstrate the robustness of CNN on regular grids, though they share trade-offs in resolution and computational cost. Graph-based approaches, by contrast, operate directly on irregular molecular structures, preserving topological information. Son [42] employed GCNs to explicitly model amino acid interactions, achieving significant accuracy gains by capturing spatial adjacency more naturally than voxel discretization. Extending geometric deep learning beyond Euclidean grids, Igashov [43] introduced Spherical CNNs to process spherical protein surface data, offering a rotation-equivariant alternative that improves robustness and precision in surface feature and binding site prediction.

Recent studies further show that structure-aware and geometric deep learning are increasingly important for biomolecular interaction and drug discovery tasks. For example, neighborhood-level structural representation learning has been used to predict lncRNA-miRNA interactions in heterogeneous biological networks [44], and geometric deep learning over heterogeneous information networks has been applied to drug repositioning [45]. In addition, DeepHIV demonstrates the utility of sequence-based deep learning for HIV-1 protease cleavage-site prediction [46], whereas MolVisGNN integrates 3D molecular spatial visual information with multi-perspective molecular representations for drug discovery [47]. These studies are related to our work at the level of structure-aware representation learning, but they address different biological tasks and input modalities. This progression from voxel grids to graphs and spherical representations reflects a broader trend: moving from brute-force discretization toward structure-aware, geometrically principled architectures that better respect protein. However, most existing methods based on GCNs suffer from two major limitations. First, many approaches fail to effectively incorporate spatial directional information, making them inadequate for capturing the complex anisotropic interactions within molecular structures. Second, most models operate on coarse-grained residue-level features, which limits their ability to capture fine-grained atomic-level interactions and thereby constrains prediction accuracy and generalization performance. Notably, existing methods still have two key limitations that hinder their performance. Firstly, most existing models rely solely on distance for edge features, ignoring the anisotropic nature of molecular interactions; Secondly, many models operate on coarse-grained residue graphs, losing the fine-grained atomic details necessary for precise binding site identification. In this study, we propose an Edge-aware GAT-based protein binding site predictor. The model constructs graphs at the atomic level and integrates both node (atom-level) features and edge features to enable efficient encoding and propagation of fine-grained spatial information.

## Result

### Performance evaluation on multiple binding site prediction tasks

To comprehensively evaluate our Edge-aware Graph Attention Network model, we employed four standard evaluation metrics: Accuracy, F1-score, Matthews Correlation Coefficient (MCC), ROC-AUC and PR-AUC. The model was assessed across five distinct molecular interaction categories: protein-protein, DNA/RNA, ion, ligand, and lipid binding sites. The quantitative results are summarized in Table 1.

**Table 1:** Comprehensive performance evaluation of the Edge-aware GAT model across different binding site categories.

Binding Category	Accuracy	F1-score	MCC	ROC-AUC	PR-AUC
Protein	0.933	0.771	0.677	0.930	0.882
DNA/RNA	0.911	0.512	0.525	0.933	0.765
Ion	0.872	0.449	0.464	0.841	0.698
Ligand	0.927	0.501	0.361	0.830	0.723
Lipid	0.736	0.323	0.459	0.921	0.587

To better evaluate performance on rare binding-site identification, which is important for imbalanced residue-level prediction tasks, we computed PR-AUC values for all five binding-site categories on the independent test set. The results in Table 1 show that the proposed model achieves competitive performance across the evaluated metrics, with category-dependent differences that are consistent with the underlying class imbalance. For the Protein binding category, the model obtains the strongest overall performance, including Accuracy of 0.933, F1-score of 0.771, MCC of 0.677, PR-AUC of 0.882, and ROC-AUC of 0.930. This result indicates that the model can effectively capture both positive and negative samples in a category where the class imbalance is relatively milder than in several other categories.

For the DNA/RNA binding category, the model maintains a high Accuracy (0.911) and ROC-AUC (0.933)—the highest among all categories—while achieving a moderate F1-score (0.512), MCC (0.525), and PR-AUC (0.765). The high ROC-AUC combined with moderate F1-score and PR-AUC reflects the severe class imbalance of DNA/RNA binding sites, where the model effectively distinguishes between binding and non-binding residues overall but faces challenges in balancing precision and recall for rare positive samples.

In the Ion binding category, the model shows a slight decline in most metrics compared to Protein and DNA/RNA categories: Accuracy (0.872), F1-score (0.449), MCC (0.464), ROC-AUC (0.841), and PR-AUC (0.698). These results are consistent with the inherent difficulty of Ion binding site prediction, as Ion-binding residues are often sparse and spatially dispersed, leading to lower detection performance across all metrics.

For the Ligand binding category, the model achieves a high Accuracy (0.927) and moderate PR-AUC (0.723), but a relatively low MCC (0.361) and F1-score (0.501) compared to other categories (except Lipid). This discrepancy is attributed to the severe class imbalance of Ligand binding sites and the high heterogeneity of Ligand-residue interactions, which makes it harder for the model to correctly identify true positive binding sites without introducing false positives.

The Lipid binding category presents the most challenging task, as evidenced by the lowest Accuracy (0.736) and F1-score (0.323) among all categories, along with the lowest PR-AUC (0.587). Despite this, the model still maintains a high ROC-AUC (0.921) and a moderate MCC (0.459), demonstrating its ability to distinguish between binding and non-binding residues at a holistic level, even when rare positive samples (Lipid binding sites) are difficult to detect.

Notably, the PR-AUC values across all categories follow the same trend as the F1-scores—consistently highest for Protein binding and lowest for Lipid binding—confirming that PR-AUC effectively reflects the model’s ability to detect rare positive binding sites.

### Architectural and hyperparameter analysis

To validate the architectural design of our proposed Edge-aware GAT and justify the selection of hyperparameter, we conducted a systematic analysis of key design variables. This evaluation focused on the trade-off between predictive performance, quantified by ROC-AUC, and computational efficiency, measured by inference time per protein. Furthermore, we examined how these choices support the structural inductive biases inherent in the model. Specifically, we assessed the impact of three core hyperparameters across the following range: the number of GAT layers (L∈{2,3,4,5,6}), the hidden dimensionality (d∈{16,32,48,64}), and the neighborhood radius (r∈{3,4,5,6,7} Å).

To ensure experimental consistency, all evaluations were conducted using the same PeSTo dataset split, with other parameters fixed to default values. Our results indicate that predictive performance plateaus at four GAT layers (ROC-AUC: 0.93); extending the depth to five or six layers yielded negligible improvements (<0.005) while doubling the inference latency. For hidden dimensionality, a 32-dimensional space proved optimal (0.93); a 16D configuration resulted in under-parameterization (0.89), whereas higher dimensions (48/64D) increased computational complexity without performance gains. Furthermore, a neighborhood radius of 5 Å was identified as the ideal threshold; smaller radii (3/4 Å) failed to capture critical atomic interactions (0.88/0.90), while larger radii (6/7 Å) introduced detrimental spatial noise (0.92/0.91).

**Fig 1:**
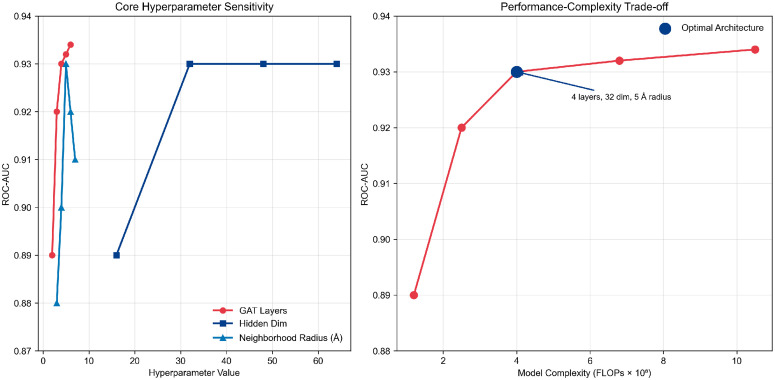
Core hyperparameter sensitivity and performance-complexity trade-off of the edge-aware GAT.

This trade-off between predictive accuracy (ROC-AUC) and model complexity (FLOPs) is illustrated in Fig 1. The results demonstrate that our final architecture—comprising 4 layers, a 32-dimensional hidden space, and a 5 Å radius—occupies the optimal Pareto point. Beyond this configuration, computational costs increase disproportionately to performance; for example, a 6-layer model requires 3.2 × more FLOPs to achieve a marginal ROC-AUC increase of only 0.004. These findings confirm that our architecture strikes a parsimonious balance between efficiency and effectiveness.

Finally, we examined the role of structural inductive biases in the proposed framework. Our edge-aware mechanism encodes Euclidean distance and unit direction vectors as geometric edge attributes that modulate the attention coefficients. However, the current architecture does not implement a strictly SE(3)- or E(3)-equivariant update rule, does not update atomic coordinates, and does not maintain explicit vector or tensor hidden channels. Therefore, this module should be interpreted as a geometry-aware and direction-informed attention mechanism rather than a rotation-equivariant tensor propagation module. The targeted ablations reported below focus on the contributions of distance features, direction vectors, and residue-level attention pooling.

### Visual analysis of predicted binding sites

To further validate the model’s capability in identifying protein binding sites, we conducted extensive visual analysis across five molecular interaction categories. The predicted high-probability binding regions were visualized using heatmaps overlaid on protein tertiary structures. Specifically, we encoded each atom’s predicted binding probability into the B-factor field of the corresponding PDB file, enabling 3D heatmap visualization of potential interaction sites using PyMOL, where red indicates high binding probability and green indicates low probability.

For enhanced biological interpretability, we aggregated atoms with predicted binding probabilities above a threshold of 0.5 into residue-level binding sites and exported the results as CSV files. Each file includes chain ID, residue index, residue name, and mean binding probability, facilitating downstream functional annotation and comparative analysis.

### Case studies of representative protein complexes

To demonstrate the model’s predictive capability and spatial localization accuracy, we conducted a series of detailed case studies on representative protein complexes across different interaction categories. For each case, the predicted binding probabilities were encoded into the B-factor field of the corresponding PDB file, enabling the visualization of high-probability interaction sites as 3D heatmaps using PyMOL. In these visualizations, red regions indicate a high binding probability, while green regions denote a low probability, providing an intuitive assessment of the predicted interfaces. The following analyses present specific predictions for protein-protein, protein-nucleic acid, ion, ligand, and lipid binding sites, comparing model outputs with known structural data.

**Fig 2:**
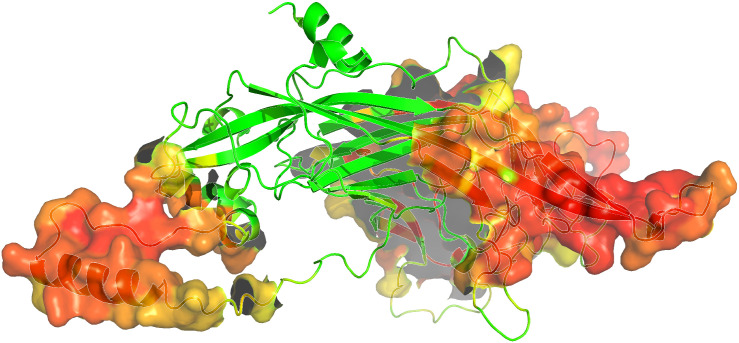
Visualization of predicted protein-protein binding sites for PDB structure 1DZL_A. Red regions indicate high binding probability.

**Protein-protein interaction:** As shown in Fig 2 and Table 2, the binding site prediction for PDB structure 1DZL_A demonstrates the model’s precision in identifying interfacial residues. The 3D heatmap shows concentrated red regions at the known interaction interface. Correspondingly, the model successfully identified key interfacial residues with high confidence, including ARG41 (0.9459), LEU61 (0.9391), and LEU43 (0.9341).

**Table 2:** High-confidence protein binding sites predicted for 1DZL_A.

chain	residue_id	residue_name	mean_probability
A	41	ARG	0.9459
A	61	LEU	0.9391
A	43	LEU	0.9341
A	23	SER	0.9218
A	64	LYS	0.9190
A	49	TYR	0.9172
A	60	ILE	0.9162
A	63	PRO	0.9053
A	34	TYR	0.8958
A	53	LYS	0.8925
A	32	ASN	0.8895
A	36	HIS	0.8864

**Fig 3:**
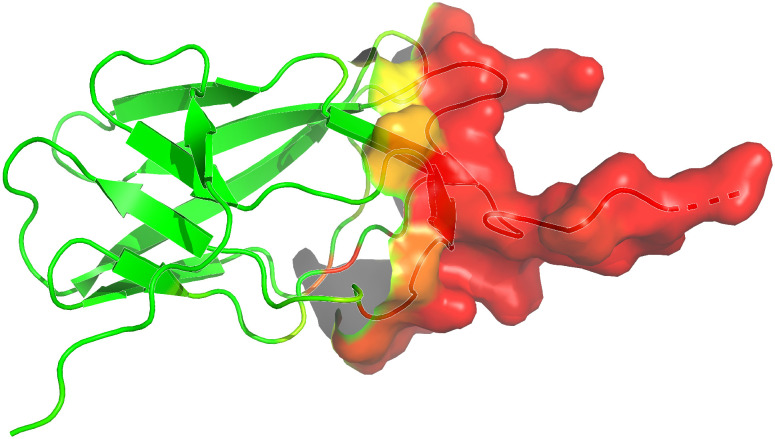
Visualization of nucleic acid binding sites for PDB structure 1H9D_A.

**Protein-nucleic acid interaction:** Fig 3 and Table 3 illustrate the prediction for nucleic acid binding sites in PDB structure 1H9D_A. The model assigned high probabilities to residues critical for nucleic binding, such as LEU71 (0.9945), PHE70 (0.9826), and ASP66 (0.9558), aligning with the expected interaction region.

**Table 3:** Predicted nucleic acid binding sites for 1H9D_A.

chain	residue_id	residue_name	mean_probability
A	71	LEU	0.9945
A	70	PHE	0.9826
A	66	ASP	0.9558
A	62	LEU	0.9558
A	65	THR	0.9354
A	58	HIS	0.9119
A	59	PRO	0.8847
A	63	VAL	0.8224
A	67	SER	0.5176

**Fig 4:**
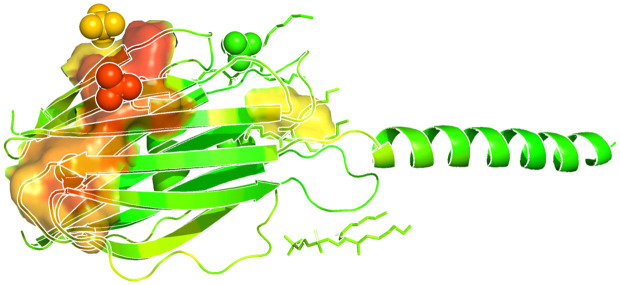
Visualization of ion binding sites for PDB structure 4TSY_B.

**Ion binding sites:** For PDB structure 4TSY_B, the predicted ion binding sites are visualized in Fig 4 and Table 4. The model localized the binding region, identifying residues like GLY27 (0.8402), VAL29 (0.8171), and LEU14 (0.7555) as the key coordination site.

**Table 4:** Predicted ion binding sites for 4TSY_B.

chain	residue_id	residue_name	mean_probability
B	27	GLY	0.8402
B	29	VAL	0.8171
B	14	LEU	0.7555
B	9	ILE	0.7514
B	30	LYS	0.7398
B	10	ASP	0.7199
B	25	ALA	0.6785
B	15	GLY	0.6730
B	28	ASN	0.5241

**Fig 5:**
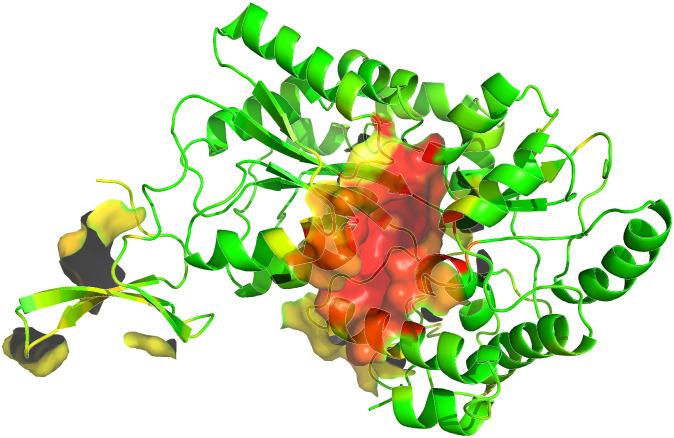
Visualization of ligand binding sites for PDB structure 5B3Z_A.

**Ligand binding sites:** Fig 5 and Table 5 present the ligand binding site prediction for PDB structure 5B3Z_A. The heatmap clearly demarcates the binding pocket, and the model pinpointed central interacting residues, notably ARG18 (0.9819), GLU32 (0.9727), and ASN23 (0.9714).

**Table 5:** Predicted ligand binding sites for 5B3Z_A.

chain	residue_id	residue_name	mean_probability
A	18	ARG	0.9819
A	32	GLU	0.9727
A	23	ASN	0.9714
A	36	GLY	0.9350
A	6	PRO	0.9116
A	30	GLN	0.8912
A	12	MET	0.8876
A	35	SER	0.8327
A	7	GLY	0.8275
A	46	TRP	0.8219
A	22	PHE	0.8076
A	10	LYS	0.7337
A	45	ILE	0.6961
A	31	TRP	0.5868
A	25	ILE	0.5774

**Fig 6:**
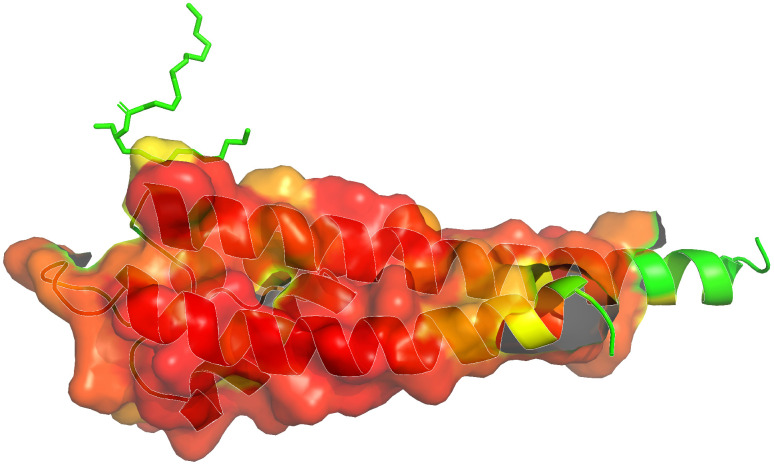
Visualization of lipid binding sites for PDB structure 6NFU_C.

**Lipid binding sites:** Finally, the lipid binding site prediction for PDB structure 6NFU_C is shown in Fig 6 and Table 6. The visualization reveals a surface patch likely involved in membrane interaction, with high-probability residues including LEU24 (0.9620), ARG27 (0.9403), and VAL34 (0.9257).

**Table 6:** Predicted lipid binding sites for 6NFU_C.

chain	residue_id	residue_name	mean_probability
C	24	LEU	0.9620
C	27	ARG	0.9403
C	34	VAL	0.9257
C	33	THR	0.8998
C	35	LEU	0.8976
C	28	ALA	0.8802
C	25	HIS	0.8796
C	26	TRP	0.8574
C	22	SER	0.8301
C	30	GLY	0.8140
C	29	ALA	0.8096
C	32	ALA	0.7953

### Web-based prediction system implementation

**Fig 7:**
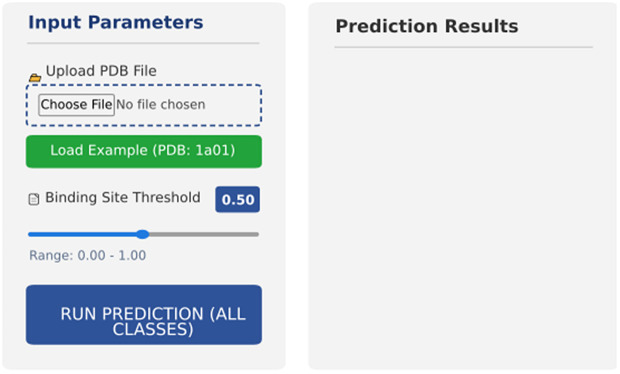
Web interface of the Edge-aware GAT prediction server.

To make our Edge-aware GAT model accessible to the broader research community, we developed an interactive web-based prediction platform (Fig 7), publicly accessible at http://119.45.201.89:5000/. The system is designed to provide researchers with an efficient and user-friendly tool for predicting protein binding sites across five molecular categories: protein-protein, DNA/RNA, ion, ligand, and lipid interactions.

**Fig 8:**
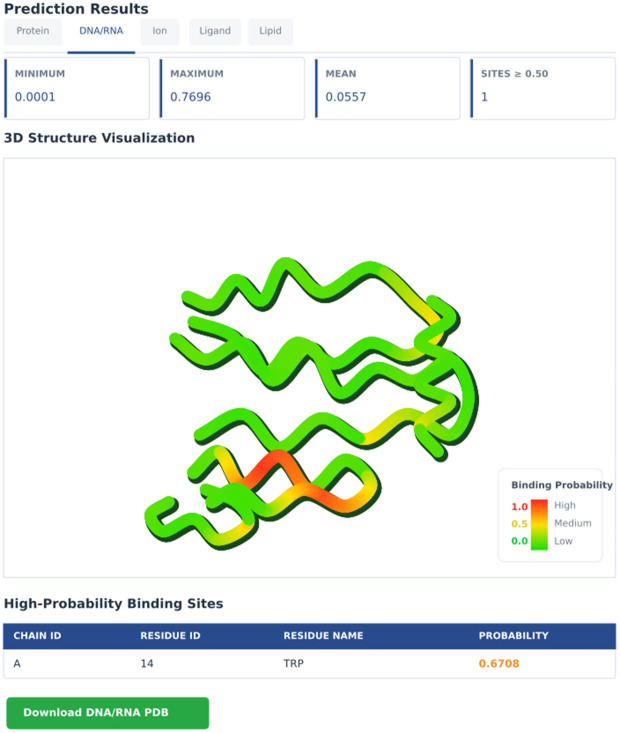
Prediction results panel.

Users begin by uploading a protein structure file in PDB format via the “Upload PDB File” option. Alternatively, an example sample structure (PDB: 1a01) may be loaded using the “Load Example” button. Following file input, the “Detect Chains” button enables the selection of specific polypeptide chains for analysis. The binding site prediction threshold is then adjustable via a slider labeled “Binding Site Threshold”, which spans a continuous range from 0 to 1, with a default preset value of 0.5. Upon configuring these parameters, users initiate the computational prediction by clicking the “RUN PREDICTION (ALL CLASSES)” button. The system performs simultaneous multi-category binding site prediction within a single inference run. For typical proteins consisting of 200–500 residues, the prediction process is completed within 10–15 seconds, ensuring real-time usability for interactive analysis. The results are subsequently displayed in the “Prediction Results” section of the interface (Fig 8).

The results are organized into several key sections. A summary panel lists positional predictions for interactions with various biomolecular classes, including protein-protein, DNA/RNA, ion, ligan, and lipid binding sites, accompanied by a corresponding legend for interpretation. A dedicated “3D Structure Visualization” panel provides a graphical representation of the protein, employing an intuitive color gradient to represent binding probabilities: green (0.0-0.3), yellow (0.3-0.7), and red (0.7-1.0). Furthermore, a table titled “High-Probability Binding Sites” enumerates specific residues with high confidence scores, detailing the chain identifier, residue number, residue name, and its prediction probability (e.g., Chain A, Residue 14, THR, 0.6788). Finally, to facilitate further analysis, an option to “Download DNA/RNA PDB” is provided. Users can manipulate the 3D structure through standard operations and access detailed residue tables listing high-confidence binding sites, with downloadable PDB files containing probabilities encoded in the B-factor field for compatibility with standard structural biology tools.

## Discussion

This study introduces an Edge-aware GAT framework to advance the prediction of protein binding sites across diverse molecular partners. Our approach extends the PeSTo model [22] by integrating multidimensional structural features and incorporating geometry-aware edge-conditioned attention. These enhancements address limitations in capturing fine-grained local structural context within protein atomic graphs while avoiding an overclaim of strict rotation-equivariant tensor propagation.

Relative to these broader graph-learning approaches [33–35], our work focuses on the finer structural scale of protein atoms and directly injects pairwise distances and direction vectors into the attention mechanism, thereby transferring the topology-attribute integration principle to 3D molecular geometry.

We integrated comprehensive structural descriptors—including secondary structure and relative solvent accessibility—with atomic-level geometric features to construct highly informative residue embeddings. This enriched feature representation provides stronger discriminative signals for identifying functional sites compared to using coordinate data alone. Furthermore, we replaced PeSTo’s global interaction modeling with sparse, edge-aware graph attention mechanism. By explicitly encoding pairwise Euclidean distances and unit direction vectors into the attention coefficients, the model incorporates local geometric context into neighborhood aggregation. Rather than enforcing strict rotation equivariance, this mechanism uses geometric edge features to bias attention toward spatially relevant atomic neighbors, thereby improving the representation of local interaction environments. This allows the network to better capture the complex spatial constraints governing molecular interactions. Finally, we incorporated a learnable query vector into the attention-based residue pooling mechanism to adaptively focus feature aggregation on functionally relevant regions. This approach enhances state consolidation, providing a more robust representation for downstream binding site prediction.

**Fig 9:**
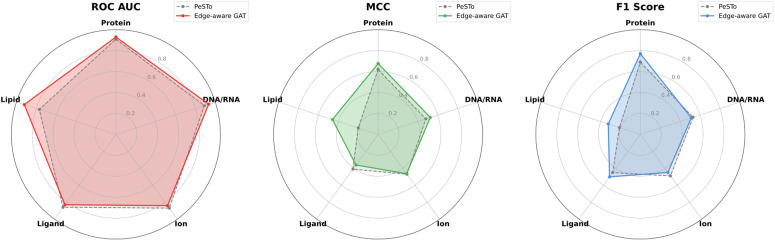
Combined radar charts of F1-score, MCC, and ROC-AUC.

Since our task involves residue-level binary classification, we discretize the predicted probabilities into binding and non-binding labels using a fixed threshold of 0.5. This choice is strategically principled to address the inherent class imbalance between spare positive binding sites and predominant negative residues. By maintaining a threshold of 0.5 across all five interaction types, we achieve an optimal balance between the recall of rare binding events and the precision of non-binding predictions. Deviating from this value would compromise model utility: a lower threshold would increase sensitivity at the expense of excessive false positives, while a higher threshold would prioritize precision at the cost of significant under-detection and a sharp decline in recall. Furthermore, adopting a 0.5 threshold aligns with the standard interpretation of sigmoid activation outputs in multi-label classification, ensuring methodological consistency and facilitating a fair, reproducible comparison with baseline models. As demonstrated in the combined radar chart panels in Fig 9, our model achieves a balanced and significant performance gain across all five biomolecular interaction categories compared to the original PeSTo framework. Notably, for protein-protein interactions, accuracy improved from 0.89 to 0.93 and the F1-score increased from 0.69 to 0.77, indicating more precise and balanced predictions. In the challenging lipid-binding category, performance gains were substantial, with the F1-score rising from 0.21 to 0.32 and the Matthews Correlation Coefficient (MCC) improving from 0.20 to 0.46, reflecting a marked reduction in both false positives and false negatives. While a minor fluctuation was observed in the ROC-AUC for ion binding (0.83 vs. PeSTo’s 0.86), the overall performance profile remains robust. These results confirm that incorporating edge geometry and directional features, coupled with attentive pooling, yields more informative residue representations and enhances generalization across varied interaction types.

The effectiveness of our framework is further validated through comparison with established baseline methods, as well as two stronger, model-centric geometric learning baselines (as recommended) for protein-protein binding site prediction. All comparative experiments were conducted under the identical PeSTo dataset, data splitting protocol, feature set and evaluation metrics to ensure strict fairness. The added geometric baselines include: (1) E(3)-equivariant GNNs (EGNN), a typical geometric architecture that explicitly models 3D geometric transformations and rotation equivariance with strong spatial representation capabilities for biomolecular structure analysis; (2) AtomGNN, a state-of-the-art surface-free atomic-level model published in 2024 that focuses on atomic-level graph construction (consistent with the core design of our method) and avoids complex protein surface parameterization.

**Fig 10:**
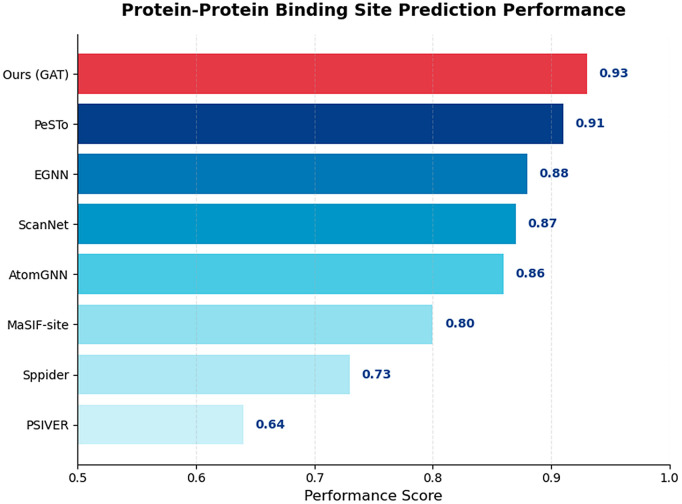
ROC-AUC performance comparison of the Edge-aware GAT model against established baseline methods.

As shown in Fig 10, our model achieves ROC-AUC of 0.93, outperforming not only traditional baselines including PeSTo (0.91), ScanNet (0.87), MaSIF-site (0.80), Sppider (0.73), and PSIVER (0.64), but also the advanced geometric learning baselines EGNN (0.88) and AtomGNN (0.86). We further performed two-tailed t-tests on the performance results, and the performance gains of our model over EGNN and AtomGNN are statistically significant (p < 0.05), demonstrating the effectiveness of the geometry aware edge attention mechanism under the evaluated benchmark.

This advantage stems from our model’s capacity to leverage both atomic-level spatial graphs and geometric edge attributes to precisely model interaction interfaces. In contrast, while PeSTo utilizes atomic geometry, its conservative interaction scoring may limit sensitivity; methods like ScanNet and MaSIF-site, which rely on specific surface representations or feature schemes, may lose fine-grained atomic details; sequence-based predictors like Sppider and PSIVER underscore the indispensable value of explicit 3D structural information. Even for geometric learning baselines (EGNN and AtomGNN) that excel in spatial representation, EGNN’s focus on global rotation equivariance neglects the fine-grained edge-level geometric attributes of binding interfaces, while AtomGNN lacks an attention mechanism to prioritize critical spatial relationships between atoms at interaction sites. Our Edge-aware GAT effectively bridges this gap by operating directly on atomic graphs while preserving and propagating critical spatial relationships through an attention mechanism, thus enabling more accurate modeling of protein binding sites.

The proposed Edge-aware GAT is also related to recent structure-aware and geometric deep learning studies in biomolecular prediction [44–47]. These works show that heterogeneous biological networks, neighborhood-level structural representations, sequence-aware predictors, and 3D molecular spatial representations can improve different biomolecular interaction or drug discovery tasks. Compared with these methods, our model is designed for atomic-level protein binding site prediction and focuses on incorporating pairwise geometric edge features into attention-based neighborhood aggregation. A natural future direction is to combine the current geometry-aware attention module with protein language model representations or strict equivariant message passing.

Nevertheless, certain limitations persist. Model performance can be influenced by the quality of input protein structures, and reliance on predicted models may introduce uncertainty. Future work will explore the integration of complementary sequence-derived features and evolutionary information to improve robustness against structural variation. Extending the model to predict other functional sites, such as post-translational modification loci, represents a promising direction for broadening its utility. Multi-task learning strategies, as employed in related works, could also be adopted to enhance overall generalization.

Although the PeSTo-style clustered benchmark split reduces redundancy between subsets, we did not construct an additional independent homology-controlled benchmark in this study. Future work will further evaluate the model using stricter sequence-identity-based splits, such as CD-HIT- or MMseqs2-based partitioning, to assess generalization under more stringent homology-control settings.

In conclusion, by integrating multidimensional structural features, geometry-aware edge attention, and residue-level attention pooling, we have developed a structure-based atomic-level framework for protein-binding site prediction. Under the evaluated benchmark protocol, the model achieves competitive performance among the compared structure-based atomic-level methods and provides interpretable spatial evidence for predicted binding regions. Future work will investigate stricter homology-controlled evaluation, protein language model-based representations, and equivariant message passing to further improve generalization.

## Methods

### Data processing

To ensure reproducibility and comparability with PeSTo and related structure-based baselines, we followed the PeSTo-style clustered benchmark split rather than constructing a new random split. The original PeSTo dataset was composed of biological assemblies from the Protein Data Bank, and protein subunits were clustered with a maximum sequence identity of 30% between clusters. As summarized in Table 7, the resulting clusters were divided into approximately 70% training, 15% validation, and 15% testing subsets. In the PeSTo benchmark, these subsets contain 376,216 training chains, 101,700 validation chains, and 97,424 testing chains, respectively. The testing set contains clusters including the 53 subunits from the MaSIF-site benchmark dataset and 230 structures from the Protein-Protein Docking Benchmark 5.0 dataset; an additional subset of 417 structures common to the ScanNet benchmark and the PeSTo testing set was used for comparative evaluation. Therefore, our evaluation follows the same redundancy-reduced clustered benchmark protocol as PeSTo. We did not generate an additional independent homology-controlled split, and we therefore avoid making a stronger claim beyond the published PeSTo-style clustered split.

**Table 7:** Simplified summary of the PeSTo-style clustered benchmark split used in this study.

Split	No. of chains	Approx. proportion	Redundancy/ homology control	Benchmark role
Training	376,216	~70%	Subunits clustered with max 30% sequence identity between clusters	Model fitting
Validation	101,700	~15%	Same clustered split as PeSTo	Hyperparameter selection
Testing	97,424	~15%	Same clustered split as PeSTo	Independent benchmark evaluation; includes clusters containing MaSIF-site and PPDB5 benchmark structures

Note. The table reports the published PeSTo split-level chain counts and clustering criterion. Because no new split was generated in this study, the homology-control evidence is based on the PeSTo clustering protocol. Residue-level class imbalance is reflected by the category-specific PR-AUC results in Table 1 and is further discussed in the Results section.

Following data partitioning, we converted each protein subunit into an atomic-level graph representation. For every atom, we encoded it spatial coordinates alongside categorical attributes—specifically element type, residue type, and atom name—into high-dimension feature vectors. Spatial topology was established using a k-nearest neighbours (k-NN) scheme based on Euclidean distances. Atom pairs within a 5.0 Å cutoff were defined as potential contacts, forming the edges of the graph for subsequent message passing.

Residue-level binding annotations adhered to PeSTo’s criteria across five interaction types (proteins/nucleic acids/ligands/ions/lipids), defining binding sites as residues with atoms ≤5 Å from partner-aligning with structural bioinformatics conventions.

While utilizing the same underlying dataset, we extended the feature representation by incorporating additional structural descriptors and implementing our novel edge-aware graph attention framework, as detailed in the following sections.

#### Feature extraction.

1. **Atomic-level features**

The atomic-level features of the model comprise five components: element type, residue category, atom type, secondary structure and relative solvent accessibility (RSA). The first three features -- element type, residue category and atom type – were formulated as described in [22]. To ensure reproducibility and consistency with baseline comparisons, the categorical vocabularies for element types, atom types, and residue categories are strictly aligned with the data processing specifications of the PeSTo paper, which we adopt as our primary dataset. For uncommon residues not covered by the standard residue category scheme, we assign them to a unified “other” category and perform one-hot encoding within the same feature coding framework, preserving their feature information without disrupting model training. Additionally, we incorporated two features: secondary structure information and RSA. The secondary structure was classified using an 8-category scheme derived from DSSP: α-helix (H), 3_10_-helix (G), π-helix (I), extended strand (E), β-bridge (B), turn (T), bend (S), and coil (C) or loop (-), and encoded as 8-dimensional one-hot vector. For missing DSSP assignments (secondary structure and RSA), we adopt a mean filling strategy based on homologous residues in the PDB dataset to complete the feature values, ensuring the integrity of multidimensional structural features as designed in our study. RSA values were normalized to range [0,1] to represent the degree of residue exposure on the protein surface.

2. **Edge features**

Edge features between connected atoms includes Euclidean distance (Eq. 1) and Unit direction vector (Eq. 2). These geometric features enable the model to capture spatial relationships and directional dependencies within the protein structure.


$$\begin{array}{c} d_{ij}=\left\|x_{i}-x_{j}\right\| \end{array}$$ (1)

$$\begin{array}{c} u_{ij}=\frac{x_{i}-x_{j}}{\left\|x_{i}-x_{j}\right\|} \end{array}$$ (2)

For alternate locations in PDB structures, we select the conformation with the highest occupancy as the only valid one for feature extraction and discard redundant low-occupancy conformations, avoiding confusion in spatial topological features during atomic-level graph construction with a 5.0 Å Euclidean distance cutoff. For non-standard PDB entries. This procedure involved completing missing backbone atoms, regularizing bond lengths and angles to standard stereochemical values, and removing solvent molecules and irrelevant heteroatoms. By only retaining the polypeptide backbone and essential side-chain atoms, we ensured that all input data adhered to a uniform specification for graph-based modeling.

#### Model architecture.

**Fig 11:**
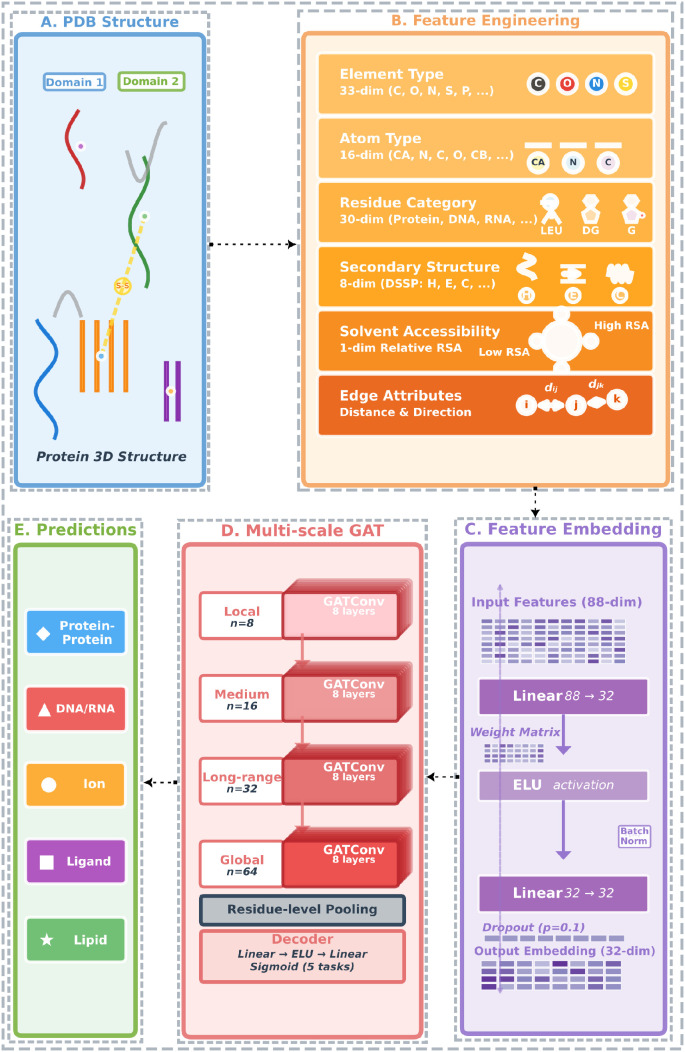
Overview of the Edge-aware GAT method. This diagram illustrates the comprehensive architecture of the Edge-aware GAT model for predicting protein binding sites. The workflow proceeds through five sequential stages: **(A) PDB Structure**. Represents the initial protein 3D structure (PDB format), often segmented into domains. **(B) Feature Engineering**. Multidimensional features are extracted. Node (atom-level) features include element type, atom type, residue category, DSSP-derived secondary structure, and relative solvent accessibility (RSA). Edge features comprise pairwise Euclidean distances and unit direction vectors between connected atoms, capturing precise spatial geometry. **(C) Feature Embedding**. The concatenated node features are projected into a compact 32-dimensional latent space via a stack of linear layers with ELU activations and dropout, generating expressive initial embeddings for graph processing. **(D) Multi-scale GAT**. The concatenated node features are projected into a compact 32-dimensional latent space via a stack of linear layers with ELU activations and dropout, generating expressive initial embeddings for graph processing. **(E) Prediction**. Outlines the final output categories for protein binding site predictions, including Protein-Protein, DNA/RNA, Ion, Ligand, and Lipid interactions. The architecture integrates atomic-level spatial graphs with directional edge attributes to model fine-grained, anisotropic molecular interactions for accurate binding sites identification.

We propose an Edge-aware Graph Attention Network (Edge-aware GAT) that operates on atomic level graphs to predict residue-level binding sites. The architecture consists of three main components (Fig 11).

1. **Feature embedding module**

Input features are projected into a 32-dimensional latent space through a stack of three fully connected layers with Exponential Linear Unit (ELU) activation functions. This embedding process generates expressive, compact representations for efficient graph processing.

2. **Edge-aware graph attention layers**

The core of our model employs 4 graph attention layers that explicitly incorporate edge features into the attention mechanism. For each pair of neighboring nodes i and j, the attention coefficient $a_{ij}$ is computed as:


$$\begin{array}{c} a_{ij}=\frac{\text{exp}\left(LeakyReLU\left({\vec{a}}^{T}\left[Wh_{i}\left\|Wh_{j}\right\|e_{ij}\right]\right)\right)}{\sum_{k\in N(i)}\text{exp}\left(LeakyReLU\left({\vec{a}}^{T}\left[Wh_{i}\left\|Wh_{j}\right\|e_{ij}\right]\right)\right)} \end{array}$$ (3)

where $h_{i}$, $h_{j}$ are node features, *W* is a learnable weight matrix, $e_{ij}$ represents edge features, and || denotes concatenation. The model maintains both scalar state vectors and tensors, which are synchronously updated during message passing:


$$\begin{array}{c} P_{i}^{\left(l+\text{1}\right)}=\sum_{j\in N_{\left(i\right)}}a_{ij}\cdot d_{ij}\cdot \mu_{ij} \end{array}$$ (4)

This coupled update mechanism ensures geometry-consistent information flow in both scalar and vector spaces.

It should be noted that Eqs. 3 and 4 do not define a strictly rotation-equivariant operation. In our implementation, the Euclidean distance ${\text{d}}_{\text{ij}}$ and the unit direction vector are encoded as geometric edge attributes and used to modulate the attention coefficient between connected atoms. Specifically, Eq. 3 computes an edge-aware attention score by combining the transformed source-node feature, target-node feature, and edge embedding. Eq. 4 then aggregates neighboring node features according to the normalized attention coefficients.

Therefore, the proposed module should be interpreted as a geometry-aware and direction-informed attention mechanism rather than a directional tensor propagation module. The model does not update atomic coordinates, does not maintain vector or tensor hidden channels, and does not impose SE(3)- or E(3)-equivariant constraints during message passing. The role of the direction vector is to provide local anisotropic edge information in the input coordinate frame, while the learned attention mechanism determines how strongly each neighboring atom contributes to the updated node representation.

3. **Residue-level pooling and decoder**

An attention-based pooling aggregates atomic features to the residue level. Given a pooling center vector $q_{pool}$, the attention weight $\beta_{i}$ for residue i is computed as:


$$\begin{array}{c} \beta_{i}=\frac{exp\left(q_{pool}^{T}\cdot q_{i}\right)}{\Sigma_{j=\text{1}}^{N}exp\left(q_{pool}^{T}\cdot q_{j}\right)} \end{array}$$ (5)

The pooled representations are obtained through weighted summation:


$$\begin{array}{c} q_{pooled}=\sum_{i=\text{1}}^{N}\beta_{i}\cdot q_{i} \end{array}$$ (6)

$$\begin{array}{c} p_{pooled}=\sum_{i=\text{1}}^{N}\beta_{i}\cdot p_{i} \end{array}$$ (7)

The resulting residue-level features are processed by a multi-layer perceptron decoder that outputs binding probabilities for five molecular classes using multi-label sigmoid activation.

#### Training procedure.

**Loss function:** To address class imbalance, we employ a weighted multi-label binary cross-entropy loss:


$$\begin{array}{c} L=-\frac{\text{1}}{N}\sum_{i=\text{1}}^{N}\sum_{c=\text{1}}^{\text{5}}\omega_{c}\left[y_{ic}\cdot {log}_{\sigma }\left({\hat{y}}_{ic}\right)+\left(\text{1}-y_{ic}\right)\cdot log\left(\text{1}-\sigma \left({\hat{y}}_{ic}\right)\right)\right] \end{array}$$ (8)

where σ denotes the sigmoid activation function, and class-specific weights $\omega_{c}$ are defined as:


$$\begin{array}{c} w_{c}=\lambda \cdot \frac{\text{1}-r_{c}}{r_{c}+\epsilon } \end{array}$$ (9)

Here, $r_{c}$ denotes the positive sample ratio for class c in the current batch, λ is a scaling factor, and ϵ ensures numerical stability.

**Optimization:** We train the model end-to-end using the Adam optimizer with a fixed learning rate of 1 × 10^−5^ and batch size of 8. Training proceeds for 100 epochs, with model checkpoints saved every 1000 steps. The checkpoint achieving the lowest validation loss is selected for final evaluation.

To enhance training stability, we implement a dummy forward pass for GPU memory preheating and weight initialization, particularly important for handling protein structures of varying sizes and graph topologies.

To clearly define the task of protein interaction site prediction and ensure reproducibility and comparability with existing methods, we formulate the problem in detail as follows, including inputs, outputs, labels, and evaluation protocol:

**Inputs**: Atomic coordinates, element types, and structural descriptors (DSSP, RSA) from PDB files.

**Outputs**: A probability score vector for each residue corresponding to five interaction types (Protein, DNA/RNA, Ion, Ligand, Lipid).

**Labels**: Binary labels assigned based on a 5Å distance threshold to the interacting partner, following the PeSTo protocol.

**Evaluation Protocol**: All models were evaluated using standard metrics (Accuracy, F1, MCC, ROC-AUC) on an independent test set following the PeSTo benchmark protocol.

#### Ablation experiments.

**Fig 12:**
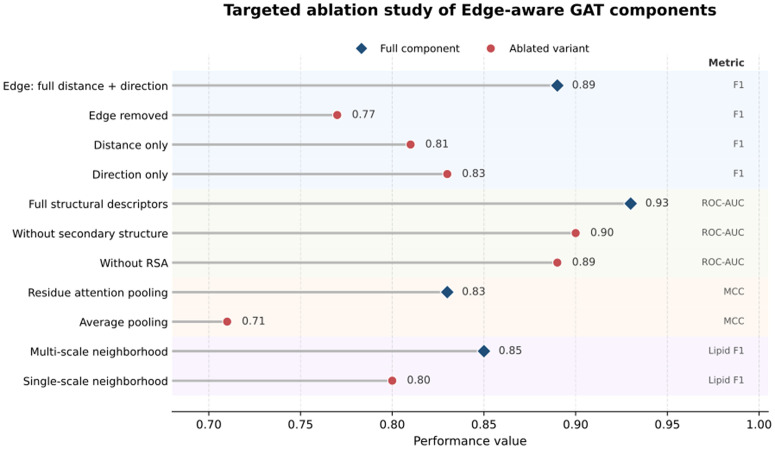
Ablation study of key components in Edge-aware GAT.

To quantitatively evaluate the individual contributions of the core components within our Edge-aware GAT framework, we conducted a series of targeted ablation experiments on the PeSTo dataset. All variants were trained and evaluated under identical protocols to ensure a rigorous comparison. As illustrated in Fig 12, the removal of the edge-aware attention mechanism resulted in the most substantial performance degradation: the complete omission of edge features reduced the F1-score from 0.89 to 0.77 (a 12% decline). Specifically, isolating either distance or directional constraints led to 8% and 6% drops, respectively, confirming that their synergistic integration is vital for capturing the fine-grained geometric interactions characteristic of binding sites.

Regarding feature engineering, the exclusion of secondary structure or Relative Solvent Accessibility (RSA) descriptors led to ROC-AUC reductions of 3% and 4%, respectively. These results underscore the importance of structural descriptors in providing complementary context for residue-level predictions. Furthermore, architectural modifications to the pooling and scaling mechanisms revealed significant dependencies: replacing attention-based pooling with average pooling decreased the MCC from 0.83 to 0.71, while reverting to single-scale neighborhood modeling reduced the lipid-binding F1-score by 5% (from 0.85 to 0.80).

By employing the metrics most sensitive to each specific functional component, these ablation results clearly delineate the drivers of our model’s performance. The edge-aware attention mechanism serves as the primary engine for encoding spatial relationships, while attention-based pooling ensures the prioritization of functionally relevant residues at the binding interface. Together, these components allow our streamlined architecture to achieve competitive performance by balancing geometric expressiveness with computational efficiency, without the need for overly rigid structural inductive biases.
